# Oral Findings, Salivary Copper, Magnesium, and Leptin in Type II Diabetic Patients in Relation to Oral *Candida* Species

**DOI:** 10.1155/2024/8177437

**Published:** 2024-07-19

**Authors:** Mohammed Jasim Mohammed, Abbas S. Al-mizraqchi, Salah M. Ibrahim

**Affiliations:** ^1^ Department of Oral Medicine College of Dentistry University of Baghdad, Bab-Almoadham, P.O. Box 1417, Baghdad, Iraq; ^2^ Department of Basic Science College of Dentistry University of Baghdad, Bab-Almoadham, P.O. Box 1417, Baghdad, Iraq; ^3^ Department of Oral Surgery College of Dentistry Kufa University, Kufa, Iraq

## Abstract

**Background:**

Type 2 diabetes is a condition in which the body becomes resistant to the effects of insulin, leading to reduced insulin production in the pancreas. It has genetic- and family-related risk factors that cannot be changed, along with modifiable lifestyle factors. The precise genetic causes of type 2 diabetes are still unknown. However, individuals can potentially slow or stop the progression of the condition by making dietary adjustments and increasing physical activity levels. *Material and Methods*. Forty-five type II diabetic patients in the study included participants between 40 and 60 years old, with a minimum duration of one year, as well as 45 healthy control subjects who were matched in terms of age and sex, and had no underlying systemic diseases. Oral examination is done for the symptoms including burning sensation, candidiasis, and a reduction in the production of saliva. The rate of saliva flow (in milliliters per minute) was measured in samples of saliva that were not stimulated. The salivary trace elements and levels of adipocytokines were evaluated using colorimetric and Ethylenediaminetetraacetic acid (ELISA) testing. The quantification of *Candida* colony numbers, an enrichment and culture approach, was used to achieve a concentration of 100,000 colony-forming units per milliliter (CFU/ml). The ShowNovo WG1 halimeter was used to measure volatile sulfur compounds in breath. The salivary glucose oxidase assay was conducted using a colorimetric technique, while the determination of trace elements was also performed using a colorimetric assay method.

**Result:**

The diabetic group exhibited a significant increase in the number of *Candida* spp colonies due to elevated levels of glucose in the saliva (*p* > 0.05). However, the variables being examined, such as body mass index (BMI), burning mouth syndrome (BMS), salivary flow rate (SFR), salivary leptin, salivary copper, and salivary magnesium, did not exhibit any significant variations in quantities between the diabetic and healthy groups (*p* > 0.05).

**Conclusion:**

The data collected in this research aid in the creation of a preventative program for oral fungal infections in individuals with type 2 diabetes. The program utilizes saliva and its constituents.

## 1. Introduction

Type 2 diabetes mellitus (DM2) is a prevalent noncommunicable disease worldwide, accounting for 90–95% of all diabetes patients. It is now considered one of the most common diseases and ranks ninth among the leading causes of death in high-income countries [1]. In addition, diabetes is linked to a higher occurrence of periodontitis. Recent investigations have also found a connection between elevated levels of MMP-8 and the impact of diabetes on their concentration in saliva [2]. Microbial biofilms cover all surfaces, both biological and non-biological, in the oral cavity. Therefore, it is crucial to maintain a balance between the host and these bacteria in order to preserve a healthy oral environment [3]. Periodontal disease and oral dryness were the primary oral symptoms observed in people with type 2 diabetes [4]. In T2DM, *β*-cell dysfunction leads to decreased insulin secretion, which prevents the body from maintaining normal glucose levels. Insulin resistance also contributes to increased liver glucose uptake and decreased glucose transport to muscle, liver, and adipose tissue. Although both factors contribute to the disease, *β*-cell dysfunction is generally more severe. However, the problem is that the coexistence of insulin resistance leads to hyperglycemia, which contributes to the progression of T2DM [1–5]. The adverse effects of hyperglycemia extend to many organs in the body, including the heart, blood vessels, eyes, kidneys, muscles, and even the bones, with potentially serious complications [6]. Patients with DM2 frequently exhibit altered magnesium (Mg) status, with Mg deficiency being more common in poorly controlled glycemic profiles and chronic complications [7]. Leptin is a hormone synthesized primarily in the small intestine [8]. Leptin, by reducing its accumulation, acts on cell receptors in the arcuate and ventromedial nuclei of the hypothalamus as well as on dopaminergic neurons within the hypothalamus and ventral tegmental area. These interactions ultimately influence feeding behavior [9]. Diabetics who consume *Candida* consume more than nondiabetics due to factors that promote oral *Candida* carriage. Many are highly sensitive. Factors can increase *Candida* species colonization in the oral cavity, such as hypopolarization, which reduces salivary flow [10]. Type II diabetes most commonly treated with metformin and Glibenclamide are examples of these antidiabetic agents [11]. Type 2 diabetes mellitus (T2DM) has a notable effect on the overall inflammatory condition of the body, the functioning of small blood vessels, and the immune system's ability to respond, thereby raising the vulnerability to infections. Type 2 diabetes mellitus (T2DM) triggers a condition of persistent, mild inflammation, resulting in disturbances in metabolism and impaired functioning of the immune system [12]. The development of *Candida* species infections, especially in the oral cavity, is strongly associated with local risk factors such as the condition of oral hygiene and the presence of removable dentures. Research has indicated that individuals who wear dentures are at a significant risk of getting *Candida*-associated denture stomatitis (CADS) because the normal *Candida* spp in their mouths might transform into harmful pathogens when certain conditions are present [13]. This study was prepared to explore the effect of age, gender, salivary Leptin, Salivary Copper and Magnesium, BMI, Salivary Glucose, and HbA1c on salivary *Candida* count by the CFU/ml method and to determine if they cause BMS, halitosis, and hyposalivation of type 2 diabetics and to compare the results with nondiabetic subjects. Saliva is considered noninvasive, less traumatic, and noncomplicated and does not require expensive tools for collection that can be used by medical service providers and diabetes care centers as a means of obtaining a complete analysis of oral conditions associated with diabetes instead of blood samples.

The Research Ethics Committee of the University of Baghdad, specifically the Faculty of Dentistry, is registered under the number 427. Every participant completes a case sheet questionnaire.

This study focuses on burning mouth sensation. All participants provided informed consent, freely and voluntarily, without any pressure, bias, or influence from the medical services provided to them. This study aims to assess oral symptoms, including burning mouth feeling, reduced salivary secretion, and volatile sulfur compounds in breath (VSCs), in individuals diagnosed with type II diabetes. Furthermore, the objective was to assess the concentrations of Copper and Magnesium, as well as Leptin and glucose in saliva, and examine their correlation with the number of *Candida* spp colony-forming units per milliliter (CFU/ml), while comparing them to a group of control participants.

## 2. Study Hypothesis

### 2.1. Null Hypothesis

There are no statistically significant differences between the presence and distribution of Salivary Magnesium and Copper, Salivary Leptin and Salivary glucose, BMI and HbA1c (as salivary biomarkers), and Salivary *Candida* count and correlation with the development of BMS, Halitosis, and hyposalivation in patients with type II diabetes mellitus.

### 2.2. Alternative Hypothesis

There are statistically significant differences between the presence and distribution of Salivary Magnesium and Copper, Salivary Leptin and Salivary glucose, BMI and HbA1c (as salivary biomarkers), and Salivary *Candida* counts, and correlation with the development of BMS, Halitosis, and hyposalivation in patients with type II diabetes mellitus.

## 3. Materials and Methods

### 3.1. Subjects

The study sample consisted of ninety subjects; they were divided into two groups:

1. Group 1: This group consisted of 45 patients, aged between 40 and 60, who had type II diabetes, and were free from any systemic diseases.
2. Group 2: In this group, forty-five control subjects who were in good health and exhibited no signs or symptoms of systemic diseases were carefully selected to match the gender and age of the patients.

The recruitment of patients was carried out at The Endocrinology and Diabetes Specialized Center situated in Al-Samawa City. All laboratory procedures were carried out within the premises of the same hospital. The duration of this study spanned from April 17, 2022, to October 17, 2022. Data were gathered based on clinical information and medical records. The selection of subjects was random, they were outpatients visitors to the center. Data were collected according to medical records, laboratory tests, and medical interview of each participant, and any subject who was smoking, drinking, diabetes type 1, gestational diabetes, or any other systemic diseases and ages less than 40 years was excluded from this study.

### 3.2. Sample Preparation

Venous blood samples (5 ml) were collected from all subjects (after 12 h of fasting) in a sitting position utilizing a single-use syringe equipped with a 21 gauge stainless steel needle. Extra blood samples were infused with Ethylenediaminetetraacetic acid. (EDTA) tubes to determine HbA1c. The tubes were labeled with the name of the sample and the time of collection with a waterproof marker. The entire nonstimulated saliva was collected by the spitting method under standardized conditions, individuals rinsing their mouths with deionized water and waiting at least 10 minutes before submitting the sample. Subjects were instructed to fast for 12 hours prior to collection; the collection was between 9 and 11 a.m. They were asked to sit in a chair and then a stopwatch was used. The samples were collected in a structured cage and fed drip. To determine saliva volume, the elapsed sampling time was measured by measuring the volume of saliva collected in 10 minutes, which calculated the salivary flow rate in ml/min (salivary flow rate = salivary volume)./time = ml/min) [14].

### 3.3. Inclusion Criteria

All patients with type II diabetes within one year and at most 40–60 years of age under treatment regimens were healthy subjects.

### 3.4. Exclusion Criteria

The study eliminated any participants who displayed the conditions stated below. Individuals who are younger than 40 years old or older than 60 years old, those with type I diabetes, gestational diabetes, thyroid and parathyroid illness, autoimmune diseases, undergoing chemotherapy, smokers, drinkers, and individuals with neoplastic diseases. Each individual provided informed consent and ethical approval was received from the Research Ethics Committee of the University of Baghdad, Faculty of Dentistry under No. 427. Every participant completes a case sheet questionnaire.

### 3.5. Intraoral Examination

In order to minimize the risk of overlooking any diseases during an examination of the inside of the mouth, it is important to systematically and consistently examine the oral cavity and its surrounding structures. The upper and lower lips should be prioritized before the buccal mucosa and gingiva. Inspect the hard palate, floor of the mouth (FOM), and oral tongue. Examine the back of the throat, the soft part of the roof of the mouth, the tonsils, the part of the throat behind the mouth, and the bottom part of the tongue using a dental mirror. In order to identify and document any oral indications, any notable changes in the mucous membranes (such as redness, swelling, ulcers, and white lesions) were observed and documented [15]. During an intraoral examination of the tongue, several phases are crucial. First, visual inspection is essential to observe any changes in the tongue's lining, such as redness, coating variations (yellow, greasy), or unusual colors like pale, purple, white, or grayish-black [16, 17]. Second, palpation helps in detecting any abnormalities like growths, firmness, tenderness, or texture changes.[18].

### 3.6. Determination of Salivary Copper and Magnesium

**Figure 1:**
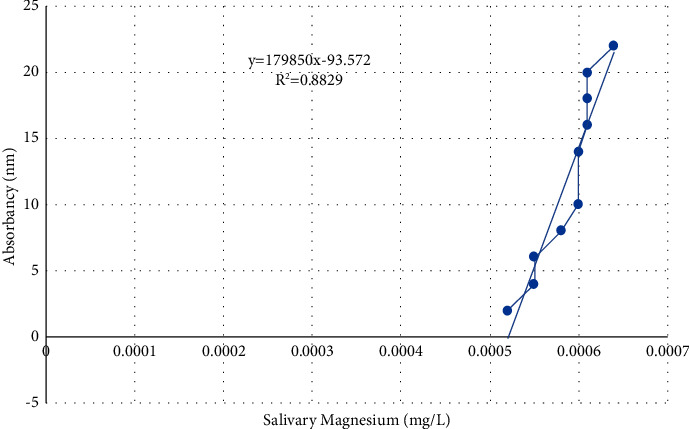
Magnesium standard curve.

**Figure 2:**
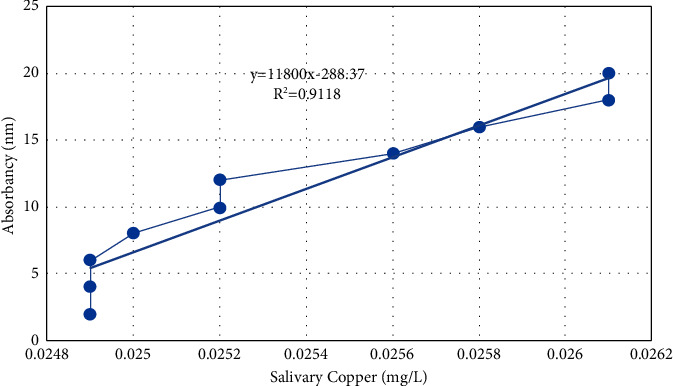
Copper standard curve.

The numbers were obtained using quantitative colorimetric measurements of metallic ions using the Di-Br-PEASA technique, a colorimetric copper reagent. The 3,5-Di-Br-PEASA chromogen undergoes a chemical reaction with ions, resulting in the formation of a colorful molecule. The intensity of this color is directly proportional to the concentration of trace elements present in the sample. Deproteinization of the saliva or blank samples is not necessary for this procedure. The reagent, distilled water (66 *μ*l), standard (66 *μ*l), and sample (66 *μ*l) were combined and left to stand for 10 minutes. Subsequently, the absorbance was measured at 560 nm using the blank as a reference. The hue remains constant for a duration of 30 minutes. Figures 1 and 2 display the standard curves for copper and magnesium.

### 3.7. *Candida* Counting (CFU/ml) Calculation

The culture medium was produced by combining 60 grams of Sabouraud Dextrose Agar (SDA) powder with 1 liter of distilled water in a sterile graduated flask. The mixture was briskly agitated to obtain a complete homogenization. The medium was heated until it entirely dissolved and reached its boiling point, and then it was autoclaved at 121°C for 15 minutes. Once the medium was cooled to a temperature ranging from 45 to 50°C, it was transferred into petri dishes or tubes to create slants. In order to improve the specificity, a concentration of 40 mg/L of streptomycin was introduced into the SDA medium [19]. Afterwards, the culture plates or tubes were placed in an environment with oxygen and kept at a temperature of 37°C for a period of 24 to 48 hours [20]. Regular inspections were conducted on a daily basis to evaluate the yeast count on the inoculation plates. The identification of *Candida* colonies was based on their visual characteristics, which included a white color, raised structure, creamy texture, and smooth surface [21]. Gram-stained sample preparations were utilized to detect *Candida* yeast cells for subsequent verification. *Candida* commonly appears as Gram-positive yeast cells (blastoconidia) and pseudohyphae with distinct constrictions in Gram-stained smears. In order to do this, a minute portion from the secluded community was utilized to generate a solution. The suspension was dried at ambient temperature and immobilized by repeatedly exposing the glass slide to the heat of a Bunsen burner. The slide was dyed using Gram's method [22]. The existence of creamy white colonies indicated the presence of *Candida*. The utilization of Gram staining and the investigation for ovoid yeasts reaffirmed this fact one again [23].

### 3.8. Salivary Leptin Assay

The saliva samples were defrosted at ambient temperature in order to quantify salivary adipocytokines using an enzyme-linked immunosorbent assay (ELISA) according to the instructions provided by the manufacturer [24]. Every saliva sample was evaluated under identical experimental settings without any dilution [25]. Initially, 50 *μ*l of standard and streptavidin-horseradish peroxidase (HRP) were introduced into the samples. Subsequently, 40 *μ*l of the sample and 10 *μ*l of Adiponectin antibodies (ADP Ab) were combined with 40 *μ*l of streptavidin-HRP, followed by incubation at 37°C for 60 minutes. After this stage, the washing solution was diluted (30X) with distilled water for future utilization. The seal plate membrane was extracted, the liquid was drained meticulously, and then the washing solution was added to each well at 30-second intervals before incubation at 37°C for 60 minutes. Following the incubation, the plates were blotted and subsequently incubated at 37°C. Gentle shaking was used to facilitate mixing. Following a 10-minute incubation at 37°C without light for color development, 50 ul of stop solution was added to each well, resulting in an immediate transition from blue to yellow hues within 10 minutes after adding stop solution; The absorbance optical density (OD) at a wavelength of 450 nm was subsequently determined within this same period and concentration calculations derived using linear regression equation derived from standard concentrations and their respective OD values were conducted.

### 3.9. Assay for Volatile Sulfur Compounds (VSCs) in Breath

The Show NOVO altimeter device was triggered by pressing and subsequently ejecting the top magazine. Upon pressing the power button for a duration of 3 seconds, a subsequent countdown of 9 seconds commenced, signifying the initiation of the preheating stage. After the preheating process finished, the gadget transitioned onto the detection stage, which was indicated by both vibration and the intermittent illumination of a blue light. It is crucial to observe that the halimeter must be utilized at a minimum of 30 minutes after using toothpaste, mouthwash, or breath fresheners in order to prevent any chemical interference that may affect the accuracy of the instrument. During the testing procedure, the patient was instructed to breathe in through their nostrils and breathe out through their mouth for a duration of 5 consecutive seconds, while keeping a distance of 1 centimeter from the breathing hole of the device. To perform a retest, simply push the power button briefly to start the procedure. The levels of volatile sulfur compounds (VSCs) are indicated by different colored lights: a green light with a slight tone represents level 1, with VSCs ranging from 0.1 to 0.5 ppm; a yellow light with a moderate tone represents level 2, with VSCs ranging from 0.6 to 1 ppm; a pink light with a serious tone represents level 3, with VSCs ranging from 1 to 2.5 ppm; and a red light indicates level 4, with VSCs exceeding 2.5 ppm, indicating bad breath.

### 3.10. Assessment of Salivary Glucose

The saliva samples were subjected to centrifugation at a speed of 14,000 revolutions per minute for a duration of five minutes in order to separate the supernatant layers. After completion, 170 microliters of 6N NaOH per milliliter of supernatant were added and mixed properly. Subsequently, another centrifugation was performed for five minutes at the same speed to obtain another supernatant, which was then analyzed using an assay factor of 1.36. Prior to commencing the test, all components were allowed to reach room temperature. The enzyme was stored in a thawed state either in the refrigerator or on ice at all times. A 300 *μ*M standard was created by combining 15 *μ*L of a 300 mg/dL solution with 818 *μ*L of deionized water. Both samples and standards were diluted by adding 20 *μ*L into separate wells. Each reaction well contained 85 microliters of Assay Buffer, 1 microliter of Enzyme Mix (vortex briefly before pipetting), and 1 microliter of Dye Reagent mixed in a clean tube. This was followed by adding 80 microliters of Working Reagent to each reaction well, which was transferred by gently tapping to ensure thorough mixing. The mixture was then incubated for 30 minutes at room temperature. Optical density readings were taken between 570–585 nm using a Cobas c 311 Photometric analyzer. Standard protocols were adjusted using five glucose solutions with concentrations ranging from 0 to 20 mg/ml. Absorbance values were measured at 540 nm to obtain precise results for each reaction well.

### 3.11. Body Mass Index (BMI)

A scale device was used with each group member to measure their weight and height in kilograms using the Quetelet equation (BMI = Mass in kg/H2 in meters) [26]. The BMI categories used include underweight (less than 18.5 kg/m^2^), normal weight (18.5 to 24.9), overweight (25–29.9), and obese (30+ kg/m^2^) [27]. Participants maintain an upright posture with their heads directed forward, excluding any factors that could lead to an increase in weight or inaccurate measurements. Weight and height are measured by manual means.

### 3.12. Assay for HbA1c and Blood Glucose

The minimum volume for the analysis of HbA1c directly from the collection tube is 1 ml of whole blood. With the selection of appropriate sample cups and software options, whole blood volumes as small as 50 *μ*L can be used. Samples collected with EDTA collection tubes were also acceptable.

### 3.13. Statistical Analysis

Statistical analysis was conducted using SPSS version 24.0. The experiments were conducted in triplicate and the findings are reported as the mean value plus or minus the standard deviation. Statistical analysis was performed using Student's *t*-test and chi-square methods, with significance evaluated at a *p* value of 0.05 or lower.

## 4. Results

### 4.1. Clinical Findings

#### 4.1.1. Gender

Of the total patient population, 26 (57.78) were male, while 19 (42.22%) were female. Similarly, among the control subjects, 26 (57.78%) were men and 19 (42.22%) were women. Interestingly, the prevalence of type II diabetes was higher among male patients compared to female patients. The percentage of male patients with type II diabetes was higher than the percentage of female patients. There were no statistically significant differences seen between the study group and the control group (*p* > 0.05) (Table 1).

#### 4.1.2. Age

Type II diabetic patients fell within the age group between 40 and 60 years, with a mean age of 51.467 ± 6.982 years and a standard deviation. Similarly, control subjects also had an Age group between 40 and 60 years. with a mean age of 49.267 ± 6.590 years and a standard deviation. After performing a *t*-test for statistical analysis, it was determined that there were no significant differences in age between patients with type II diabetes and control subjects (*p* > 0.05) (Table 2).

#### 4.1.3. Salivary Trace Elements

The mean salivary magnesium (Mg) concentration in the study group was 0.02121 ± 0.00445, while the mean concentration in the control group was 0.00062 ± 0.00006. Similarly, the mean salivary copper (Cu) concentration in the study group was 0.04836 ± 0.02206, compared to 0.02605 ± 0.00072 in the control group. The statistical analysis, conducted using a *t*-test, revealed that there were very significant differences between the groups in terms of Cu and Mg, with a *p* value of less than 0.05 (Table 3).

Measurement of Leptin in Saliva: In diabetics, the average (mean) leptin level was 7.181 with a standard deviation of 0.913, while in the control group, the average leptin level was 5.234 with a standard deviation of 0.946. The application of a *t*-test for statistical analysis revealed significantly significant disparities between the diabetic and control groups (*p* < 0.05) (Table 4).

Glucose in saliva across different groups: The collected results indicated that the average level of glucose in the saliva was 9.03 ± 1.079 mg/dl in the control group and 17.71 ± 2.087 mg/dl in the study groups. The *t* test revealed statistically significant but weak differences between groups, with a *p* value of less than 0.05 (Table 5). The collected data revealed that the average salivary glucose level was 9.030 ± 1.079 mg/dl in the control group and 17.717 ± 2.088 mg/dl in the experimental groups. The statistical study, employing a *t*-test, revealed highly significant disparities between the groups with a *p* value of less than 0.05 (Table 5).

Body mass index (BMI): The study revealed that the average and variability of BMI in diabetic patients were 33.471 ± 4.359 and 33.729 ± 4.798 respectively, compared to the control group. The application of a *t*-test for statistical analysis did not reveal any statistically significant distinctions between the diabetic and control groups (*p* > 0.05) (Table 6).

Salivary *Candida* spp count: The study revealed that the average and standard deviation of *Candida* count per milliliter of saliva were 24.718 ± 3.682 in the study group and 6.467 ± 3.520 in the control group. The application of a *t*-test for statistical analysis revealed significantly significant disparities between the diabetic and control groups (*p* < 0.05) (Table 7).

#### 4.1.4. Blood Glucose and HbA1c

The mean and standard deviation of HbA1c and blood glucose of the study group were 8.800 ± 1.814% and 184.711 ± 60.037 mg/dl, respectively, while in the control group 5.636 ± 0.403% and 103.578 ± 13.250 mg/dl, respectively. The statistical study, utilizing a *t*-test, revealed substantial and meaningful disparities between the diabetes and control groups (*p* < 0.05) (Table 8).

### 4.2. Oral Findings

#### 4.2.1. Burning Mouth Syndrome

The study showed that the number and percentage of patients complaining of burning mouth syndrome were 11 (24.44%) and 34 (75.56%) were free of symptoms, while in the control group 5 (11.11%) had symptoms and 40 (88.89%) were free. Statistical analysis using Chi-square showed nonsignificant. There were no statistically significant differences between the study and control groups (*p* > 0.05) (Table 9).

#### 4.2.2. Salivary Flow Rate

The study revealed that the average and variability of in the study group were 0.410 ± 1.134, while in the control group they were 0.636 ± 0.410. The application of a *t*-test for statistical analysis revealed significantly significant disparities between the diabetic and control groups (*p* < 0.05) (Table 10).

#### 4.2.3. VSCs in Breath

Had a mean of 0.509 ± 1.74 ppm in the control and 0.538 ± 0.204 ppm in the study group, The statistical analysis, conducted using a *t*-test, indicated that there were no significant differences between the study group and the control group (*p* > 0.05) (Table 11).

### 4.3. Correlations *Candida* CFU/ml × 10^3^ with Parameters

#### 4.3.1. Age

The study revealed a statistically significant weak positive connection between the count of *Candida* in saliva and age across all groups (*p* < 0.05) (Table 12).

#### 4.3.2. Gender

This investigation revealed a statistically significant weak negative correlation (*p* < 0.05) between the control women and the study women, as well as between the control men and the study females and study males (Table 13).

#### 4.3.3. Salivary Copper and Magnesium

The study presents the results of the correlation analysis between salivary copper and magnesium levels and the number of colony-forming units per milliliter (CFU/ml) in all groups. The analysis showed a weak negative association between salivary magnesium and copper levels and CFU/ml, but this correlation was not statistically significant (*p* > 0.05) (Table 14).

#### 4.3.4. Salivary Leptin

The study showed a modest negative connection in all groups; however, this correlation was not statistically significant (*p* > 0.05) (Table 15).

#### 4.3.5. Salivary Glucose Levels

The study showed a weak positive association with CFU in the control group, but this correlation was not statistically significant. However, in the study group, there was a weak positive correlation between salivary glucose levels and CFU, and this correlation was statistically significant with a *p* value of less than 0.05 (Table 16).

#### 4.3.6. BMI

There was a weak negative connection between the count of *Candida* spp and BMI in both the control and study groups 1 and 2, although this link was not statistically significant (*p* > 0.05) (Table 17).

#### 4.3.7. HbA1c and Blood Glucose

There were moderate positive significant correlations between *Candida* count and blood glucose and HbA1c and B. glucose in study group 1 and a strong positive significant correlation in group 2, while a weak positive nonsignificant correlation in controls *p* > 0.05 (Table 18).

### 4.4. Salivary *Candida* Count Correlation with the Oral Findings

#### 4.4.1. BMS

The results showed that CFU/ml had a weak positive but not significant correlation with BMS in all groups, *p* > 0.05 (Table 19).

#### 4.4.2. Salivary Flow Rate (SFR)

The results below show that in the control group, CFU had a weak negative significant correlation with SFR. In the study group, CFU had a weak negative correlation and a nonsignificant correlation with SFR (Table 20).

#### 4.4.3. VSCs in Breath

*Candida* count showed a moderate positive nonsignificant correlation between *Candida* count and volatile sulfur compounds in all groups *p* > 0.05 (Table 21).

## 5. Discussion

In 2015, approximately 392 million people were diagnosed with the disease, a significant increase from approximately 30 million in 1985 [28]. Although it typically occurs in middle or older age [29], rates of type 2 diabetes are also increasing in young people [30].

The study findings indicated that diabetic type II patients fell within Individuals between the ages of 40 and 60 are more susceptible to getting type 2 diabetes. This is because aging leads to higher levels of insulin resistance and reduced pancreatic islet function [31]. In terms of sex, male patients with type II diabetes outnumbered female patients. Gender differences in chronic diseases such as diabetes are influenced by a combination of biological and environmental sociocultural factors [32].

Chronic systemic inflammation associated with obesity is the result of dysfunction of the adipose tissue. Various plausible explanations for this phenomenon have been proposed, including alterations in adipokine secretion, fatty acid-induced inflammation, oxidative stress, endoplasmic reticulum (ER) stress, or hypoxia of adipose tissue, [33] and may act in concert. Whether Candida directly methylates mercaptan remains unknown. This aspect of Candida's role in oral health requires further investigation. Future studies should examine tongue coating levels and bacterial species present on the tongue, periodontal margins, and within root pockets to further elucidate the role of Candida in oral disease [34].

*Candida albicans* was the infection most frequently found in patients with BMS. However, the correlation between the number of *Candida* in saliva and the symptoms of Burning Mouth Syndrome (BMS) was not clearly established [35]. The exact explanation for the relationship between *Candida* count and BMS in diabetes type II may be due to factors such as a compromised immune system and extra sugars in yeast-friendly areas [36]. *Candida* counts increased in the diabetic groups due to decreased salivary flow rates [37], more likely to impaired salivary flow in type 2 diabetics compared to nondiabetic subjects. Another study found an association between yeast isolation and the low rate of salivary flow evaluating the presence of *Candida* species and other yeasts in the oral cavities of type 2 diabetic patients [38], since the adhesion of *Candida* species colonization is required over the mucosal surface, the washing away property of saliva prevents such adhesion, and colonization of *Candida* spp would be diminished due to a decrease in salivation in the diabetic group more than in the control [39]. The weak nonsignificant negative correlation between *Candida* spp density and salivary microbiome may reflect two distinct aspects of *Candida* commensalism: first, that the increase in salivary iron concentration of fungal increases in low levels of *Candida* and second, that high levels of *Candida* infection in the mouth destroy salivary iron because of nutritional immunity of the host or because of a trigger mechanism [40]. The inverse relationship between leptin and *Candida* count can be explained by the effect of leptin on neutrophil chemotaxis and infiltration mediated by human neutrophil migration by activating certain enzyme kinases [41]. A case-control study investigated the correlation between salivary glucose, blood glucose, and oral candidal carriage in the saliva of type 2 diabetics and found a statistically significant A significant correlation has been found between the levels of glucose in saliva and the number of *Candida* present [42]. This study proposes a potential correlation between the number of *Candida* in saliva and the levels of glucose in saliva among individuals with type II diabetes.

### 5.1. Limitations of the Study

Because of financial constraints, we had to get the fasting glucose, glycated hemoglobin, *Candida* culture, and other patient data measured at a commercial laboratory that is not affiliated with the Diabetic Center. The study participants were picked using the random consecutive sampling technique, which may introduce selection bias. Moreover, the study employed a case-control design, focusing on individuals who visited the Center as outpatients rather than the general population. It is important to note that the observed correlation between the measured parameters may not necessarily indicate a true association.

## 6. Conclusion

This study investigates the correlation between the number of *Candida* microorganisms and several oral manifestations, including symptoms of Burning Mouth Syndrome (BMS), salivary flow rate (SFR), volatile sulfur compounds (VSCs) in breath, as well as clinical factors such as age, gender, and body mass index (BMI). Furthermore, the laboratory results include measurements of salivary magnesium, salivary copper, salivary leptin, salivary glucose, HbA1c, and blood glucose. Our investigation indicates that there is no link between *Candida* CFU/ml counts and body mass index (BMI) in all categories. The findings indicate that there is no correlation between the symptoms of BM and the presence of volatile sulfur compounds (VSCs) and the concentration of *Candida* spp in the saliva, measured in colony-forming units per milliliter (CFU/ml), in both the study and control groups. However, there was no link observed between the SFR in the control group and the CFU/ml in diabetics. In fact, there was a reverse relationship between the two variables. The count of *Candida* spp was not found to be linked with the levels of salivary copper and salivary magnesium in any of the groups. The correlation between salivary leptin and CFU/ml was not observed in any of the groups. There was a link between salivary glucose and *Candida* spp count in the study group. The study group showed a direct correlation between HbA1c, blood glucose, and CFU/ml. The data collected in this study provides valuable insights for the creation of a preventative program targeting oral fungal infections in individuals with type 2 diabetes, utilizing saliva and its constituents.

## Figures and Tables

**Table 1:** Distribution of gender among groups.

		Groups		Chi-square	*p* value	Total
		Control	Study			
M	*N*	26	26	0	1.00 NS	52
	%	57.78	57.78			57.78
F	*N*	19	19			38
	%	42.22	42.22			42.22

M: male; F: female; NS: nonsignificant.

**Table 2:** The age distribution and average ages of individuals with type II diabetes.

	Min.	40.000	*T*-test	*p* value

Control	Max.	60.000		
	Mean	49.267		
	±SD	6.590		

	Min.	40.000	1.537	0.128 NS

Study	Max.	60.000		
	Mean	51.467		
	±SD	6.982		

**Table 3:** Calculating the mean and standard deviation of the salivary magnesium and salivary copper.

Groups		Mg	Cu
Study	Min.	0.00052	0.02490
	Max.	0.00070	0.02760
	Mean	0.00062	0.02605
	±SD	0.00006	0.00072

Control	Min.	0.01390	0.01280
	Max.	0.02990	0.08530
	Mean	0.02121	0.04836
	±SD	0.00445	0.02206

*T*-test^*∗*^		31.055	6.779
*p* value		**0.000**	**0.000**

^*∗*^Significant at *p* < 0.05; *p* value =0.01.

**Table 4:** Salivary leptin among groups (ng/ml).

Vars.	Groups								*T*-test	*p* value
	Control				Study					
	Min.	Max.	Mean	±SD	Min.	Max.	Mean	±SD		
Leptin	2.49	6.970	5.234	0.946	5.250	8.770	7.181	0.913	9.935^*∗*^	0.000

^*∗*^Significant at *p* < 0.05.

**Table 5:** Glucose in salivary among groups mg/dl.

	Groups		*T*-test	*p* value
	Control	Study		
Minimum	7.240	14.430	24.796^*∗*^	**0.000**
Maximum	10.680	21.440		
Mean	9.030	17.717		
±SD	1.079	2.088		

^*∗*^Significant at *p* < 0.05; *p* value =0.01.

**Table 6:** BMI among participants (kg/m^2^).

			*t*-test	*p*
Control	Min.	21.799	0.267	0.790 NS
	Max.	41.270		
	Mean	33.729		
	±SD	4.798		
Study	Min.	23.529		
	Max.	42.202		
	Mean	33.471		
	±SD	4.359		

NS: nonsignificant.

**Table 7:** *Candida* counts among groups (CFU × 10^3^/ml).

	Groups		*T*-test	*p* value
	Control	Study		
Minimum	1.000	19.000	23.323^*∗*^	0.000
Maximum	14.000	31.000		
Mean	6.467	24.178		
±SD	3.520	3.682		

^*∗*^Statistically significant at a significance level of *p* < 0.05.

**Table 8:** Levels of blood glucose (measured in milligrams per deciliter) and HbA1c (expressed as a percentage) among different groups.

Groups		HbA1c	Glucose	*T*-test	*p* value
Control	Min.	4.900	73.000	11.420^*∗*^	0.000
	Max.	6.700	127.000		
	Mean	5.636	103.578		
	±SD	0.403	13.250		

Study	Min.	6.300	80.000	8.852^*∗*^	0.000
	Max.	14.200	407.000		
	Mean	8.800	184.711		
	±SD	1.814	60.037		

^*∗*^Significant at *p* < 0.05.

**Table 9:** Burning mouth syndrome (BMS) among participants.

			*N*	%	Chi-square	*p* value
Groups	Control	With	5	11.11	2.736	0.098 NS
		Free	40	88.89		
	Study	With	11	24.44		
		Free	34	75.56		

NS: nonsignificant.

**Table 10:** Average of SFR and standard deviation of control and study.

	Control				Study				*T*-test	*p* value
	Min	Max	Mean	SD±	Min	Max	Mean	SD±		
SFR	0.460	0.700	0.636	0.044	0.41	0.6	0.41	0.133	9.040^*∗*^	0.00

^*∗*^Highly significant if *p* < 0.001.

**Table 11:** Mean and standard deviation of VSCs between control and study.

	Control				Study				*T*-test	*p* value
	Min	Max	Mean	SD±	Min	Max	Mean	SD±		
VSCs in breath ppm	0.3	0.9	0.496	0.16	0.3	0.9	0.537	0.203	0.83	0.4NS

NS: nonsignificant.

**Table 12:** Correlation between the count of *Candida* in saliva (measured in colony-forming units per milliliter) and age across different demographics.

	Salivary *Candida* count (CFU/ml)			
	Control		Study	
	*r*	*p*	*r*	*p*
Age	0.146	0.000	0.522^*∗*^	0.000

^*∗*^Significant at *p* < 0.05.

**Table 13:** The relationship between the number of *Candida* microorganisms in saliva (measured in colony-forming units per milliliter) and gender across different groups.

		Salivary *Candida* count			
		Study females		Study males	
		*r*	*p*	*r*	*p*
Salivary *Candida* count	Control females	−0.06	0.0304	−0.171^*∗*^	0.000
	Control males	−0.101^*∗*^	0.000	−0.331^*∗*^	0.000

^*∗*^Significant at *p* < 0.05.

**Table 14:** Relationships of CFU/ml × 10^3^ with the salivary magnesium and copper.

Vars.	CFU/ml × 10^3^			
	Groups			
	Control		Study	
	*r*	*p*	*r*	*p*
Magnesium	−0.173	0.255	−0.189	0.299
Copper	−0.055	0.718	−0.269	0.137

**Table 15:** Relationships of CFU/ml × 10^3^ with the salivary leptin.

Vars.	CFU/ml × 10^3^			
	Groups			
	Control		Study	
	*r*	*p*	*r*	*p*
Leptin	−0.055	0.719	−0.294	0.103(NS)

NS: nonsignificant.

**Table 16:** Relationships of CFU/ml × 10^3^ with salivary glucose.

Vars.	CFU/ml × 10^3^			
	Groups			
	Control		Study	
	*r*	*p*	*r*	*p*
Salivary glucose	0.554	0.06	0.603	0.04^*∗*^

^∗^Significant at *p*<0.05.

**Table 17:** Relationship of CFU/ml × 10^3^ with BMI.

Vars.	CFU/ml × 10^3^			
	Groups			
	Control		Study	
	*r*	*p*	*r*	*p*
BMI	−0.021	0.890	−0.114	0.456

**Table 18:** Correlation of *Candida* spp count with HbA1c and B. glucose.

	*Candida* spp count					
	Control		Study			
			Category 1 (1–10 years)		Category 2 (11–20 years)	
	*r*	*p*	*r*	*p*	*r*	*p*
HbA1c	0.409	0.07	0.613	0.05^*∗*^	0.709	0.03^*∗*^
B. glucose	0.554	0.06	0.603	0.04^*∗*^	0.716	0.029^*∗*^

^*∗*^Significant at *p* < 0.05.

**Table 19:** Relationship of salivary *Candida* spp count with BMS by groups.

	*Candida* spp count					
	Control		Study			
			Category 1 (1–10 years)		Category 2 (11–20 years)	
	Chi	*p*	*r*	*p*	*r*	*p*
BMS	2.736	0.098	0.284	0.116	0.417	0.157 (NS)

NS: nonsignificant.

**Table 20:** Relationship of salivary *Candida* spp count and SFR by groups.

	CFU/ml					
	Control		Study			
			Category 1 (1–10 years)		Category 2 (11–20 years)	
	*r*	*p*	*r*	*p*	*r*	*p*
SFR	−0.690	0.06 (NS)	−0.661	0.05	−0.723	0.03^*∗*^

NS = nonsignificant; ^*∗*^=significant at *p* < 0.05.

**Table 21:** Relationship of salivary *Candida* spp count/ml with VSCs in breath in study categories.

	C. spp count					
	Control		Study			
			Category 1 (1–10 years)		Category 2 (11–20 years)	
	*r*	*p*	*r*	*p*	*r*	*p*
VSCs	0.677	0.614 (NS)	0.684	0.611 (NS)	0.601	0.130 (NS)

NS: nonsignificant.

## Data Availability

The data created and examined during the current study can be available upon reasonable request.

## Abbreviations

MMP-8: Matrix metalloproteinase-8
VSCs: Volatile sulfur compounds
BMI: Body mass index
BMS: Burning mouth syndrome
SFR: Salivary flow rate
T2DM: Type II diabetes mellitus
EDTA: Ethylenediaminetetraacetic acid
FOM: Floor of the mouth
Di-Br-PAESA: Dibromo-paesa
SDA: Sabouraud dextrose agar
HRP: Horseradish peroxidase
ADP Ab: Adiponectin antibodies
OD: Optical density
ER: Endoplasmic reticulum.

## Additional Points

*Data Management and Analysis*: After quantitative analysis of the results of biological resistance, continuous variables are often converted into clusters, as they are much easier to analyze and make decisions.
